# Body Mass, Total Body Fat Percentage, and Visceral Fat Level Predict Insulin Resistance Better Than Waist Circumference and Body Mass Index in Healthy Young Male Adults in Indonesia

**DOI:** 10.3390/jcm7050096

**Published:** 2018-05-01

**Authors:** Liong Boy Kurniawan, Uleng Bahrun, Mochammad Hatta, Mansyur Arif

**Affiliations:** 1 Department of Clinical Pathology, Faculty of Medicine, Hasanuddin University, Makassar 90245, Indonesia; ulengbahrun68@gmail.com (U.B.); mansyurarif64@gmail.com (M.A.); 2 Department of Immunology and Biomolecular, Faculty of Medicine, Hasanuddin University, Makassar 90245, Indonesia; hattaram@yahoo.com

**Keywords:** insulin resistance, body weight, body fat, visceral fat, waist circumference, body mass index

## Abstract

The incidence of obesity which leads to insulin resistance (IR) and metabolic disorder is increasing in developing countries, including Indonesia. Male adults have a higher risk of abdominal obesity than females. This is associated with cardiometabolic disorders. Several anthropometric measurements have been proposed to predict IR. The aim of this study was to investigate whether body mass, body mass index (BMI), waist circumference (WC), body fat percentage (BF) or visceral fat level (VF) could become a better predictor of IR in healthy young male adults. A total of 140 healthy young male adults ranging from 18–25 years were recruited in the study. Insulin resistance was measured by calculating their Homeostatic Model Assessment for Insulin Resistance (HOMA-IR). Subjects with a HOMA-IR value ≥75th percentile, with cut off 3.75, were defined as IR. Anthropometric measurements including body weight, BMI, and WC were performed, whereas BF and VC were measured using bioelectrical impedance analysis (BIA). IR had a strong correlation with body weight, BMI, WC, BF, and VF. In the area under the curve of body mass, BF and VF were slightly greater than WC and BMI. Anthropometric measurements correlated strongly with IR but body weight, BF, VF had a stronger correlation than WC and BMI in healthy young male adults.

## 1. Introduction

Obesity is defined as excess or abnormal fat mass [1]. It is estimated worldwide that there are 1.2 billion overweight people with obesity affecting 300 million of them [2]. In the past 20 years, the prevalence of obesity has increased both in Indonesia and in adult males [3]. Obesity is associated with several metabolic disorders including insulin resistance (IR) and diabetes mellitus (DM). Obesity induces the development of IR through several mechanisms [4,5]. IR is a predisposing factor for the development of DM and metabolic syndrome [6,7].

The gold standard for measuring insulin resistance is the quantification method, using hyperinsulinemic normal blood glucose clamp. However, the procedure is quite difficult, inconvenient, expensive, and time-consuming to perform. A development called the homeostasis model assessment of IR (HOMA-IR) is commonly used as an alternative method to evaluate insulin resistance which is more convenient, simple, fast, and cost-effective [8,9].

Even though the causes of IR are multifactorial, obesity has a strong correlation with the development of IR. Obesity can be measured by several anthropometric properties such as body weight (BW), body mass index (BMI), waist circumference (WC), and recently obesity indices commonly measured by body fat percentage (BF) and visceral fat level (VF) performed by bioelectrical impedance analysis. WC is commonly used as the criteria for abdominal obesity as evidence shows that fat distribution in the visceral region is correlated well with IR [10].

The aim of this study was to evaluate the association between five common obesity indices including BW, BMI, WC, BF, and VF with IR, and to stratify their diagnostic value for predicting IR in a healthy young male adult population.

## 2. Material and Methods

### 2.1. Study Sample

This was a cross-sectional study performed during the period of July 2017 to February 2018. All voluntary participants were 1st to 6th grade male medical students of Hasanuddin University, Makassar, Indonesia who agreed to join the study and gave written informed consent. A total of 140 subjects were recruited to join the study. All subjects fasted overnight for at least 8 h and fasting blood was collected. Anthropometric measurements were also obtained from participants. All study samples were of Asian ethnicity and predominantly Indonesian. We excluded participants who used medication including oral hypoglycemic agents, lipid reducing drugs, and corticosteroids, and those with a history of diabetes mellitus. This study was approved by the Komite Etik Penelitian Kesehatan (Health Research Ethical Committee) in the Medical Faculty, Hasanuddin University, Makassar, Indonesia and complied with the Declaration of Helsinki.

### 2.2. Anthropometric and Laboratory Measurements

Anthropometric measurements were performed by a single examiner. BW was measured by using Tanita BC-541 (Tokyo, Japan), the height was measured by Seca stadiometer, and the BMI was calculated as weight (kg) divided by height squared (m^2^). The WC was measured at the midway level between the iliac crest and the lower border of the 12th rib using Seca measuring tape. The BF percentage and VF level were measured by using Tanita BC-541 bioelectrical impedance analysis (BIA). Blood samples were collected after an overnight fasting period of at least 8 h. Fasting glucose was measured using Abx Pentra 400 (Horiba, Edison, NJ, USA) while insulin was measured using Elecsys 2010 (Roche, Indianapolis, IN, USA).

### 2.3. Definition of IR

Insulin resistance was calculated using the homeostatic model assessment of insulin resistance (HOMA-IR) index = (Insulin (µIU/mL) × Fasting Blood Glucose (mg/dL)/405. HOMA-IR value ≥75 percentile was used as a cut-off to define IR. In our study, the cut-off value for IR was 3.75. All HOMA-IR values below the cut-off were defined as insulin sensitive/non-IR.

### 2.4. Statistical Analysis

The normality of data distribution was tested using the Kolmogorov–Smirnov test. All normally distributed data were expressed as the mean ± standard deviation (SD) while non-normally distributed data were expressed as median (minimum–maximum). The BF and fasting plasma glucose were normally distributed, while age, BW, height, BMI, WC, VF, insulin, and HOMA-IR were not normally distributed. The correlation of HOMA-IR and all variables were analyzed with the Spearman Correlation Test. Receiver operating characteristic (ROC) curves were generated for BW, BMI, WC, BF and VF as predictors of IR. The area under the ROC curve (AUC) and the optimal cut-off points for IR prediction of BW, BMI, WC, BF, and VF were determined by the largest sum of sensitivity and specificity. All statistical analyses were performed using the Statistical Package for the Social Sciences, Version 21.0 (SPSS Inc., Chicago, IL, USA). Statistical significance was defined as *p* < 0.05.

## 3. Results

The characteristics of the subjects are shown in Table 1. There is no significant difference of age between IR and non-IR Groups. BW, BMI, WC, BF, and VF are significantly higher in the IR group compared with non-IR groups. The FPG does not differ significantly between both groups but insulin and HOMA-IR are significantly higher in the IR group.

BW, BMI, WC, BF, VF show a significant correlation with HOMA-IR (Table 2).

**Figure 1:**
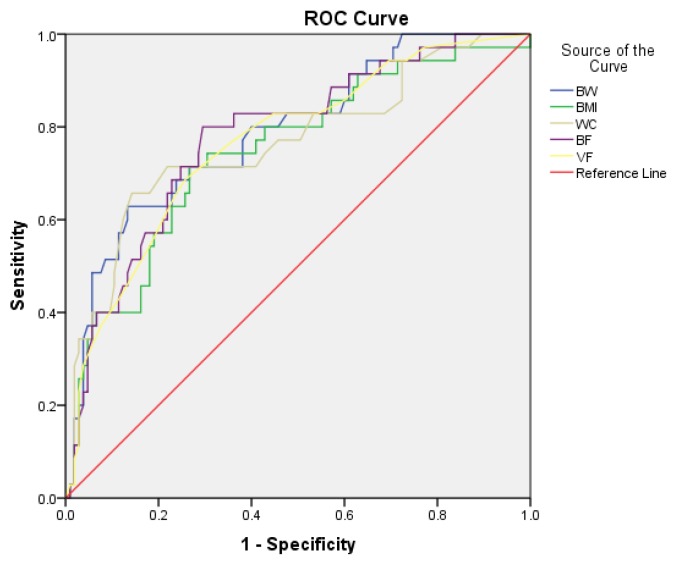
ROC Curve for BW, BMI, WC, BF, and VF as predictors of IR. IR = Insulin Resistance, BW = body weight, BMI = body mass index, WC = waist circumference, BF = body fat percentage, VF = visceral fat level, ROC = receiver operating characteristic curve.

The ROC curve shows that the AUC of BW, BMI, WC, BF, and VF has a strong prediction of IR, with BW, BF, and VF having a better prediction value than WC and BMI (Figure 1). BF has the highest sensitivity by using the cut-off 22.05 whereas WC has the highest specificity by using the cut-off 91.5 (Table 3).

Logistic regression shows every 1-point increase of BW, BMI, WC, BF, and VF increases 1.065, 1.144, 1.076, 1.155, 1.227 occurrences of IR respectively (Table 4).

## 4. Discussion

In this study of healthy young Indonesian male adults, the cut-off value of HOMA-IR was 3.75. This was higher than what was proposed by earlier studies. A Caucasian study had a cut-off of 2.29, a middle age, elderly Taiwanese study had a cut-off of 2.30, and an Iranian study had a cut off of 2.6 [11,12,13]. This difference might be due to a difference in ethnicity, gender, and age group. The results of our study show that five obesity indices, BW, BMI, WC, BF, and VF, all have a significant correlation with IR. Further analysis revealed that the AUC of BW, BF, and VF was slightly larger than WC and BMI in predicting IR while Cheng [12] reported that AUC of BMI and WC were slightly larger than BF in predicting IR. This difference could be due to the difference in age groups (50–90 years old in the Taiwanese study compared to 18–25 years old in our study. Further, our data were homogenous (male only subjects) while the Cheng [12] data were heterogeneous (male and female subjects). An interesting finding in our study was that BW was found to have a stronger correlation with IR and had the largest AUC area compared with other obesity indices, with the best cut-off being 70.20 kg in our population, as BW was rarely used as the obesity index. We proposed the BW cut-off point of 70.20 kg as a simple IR predictor for young male adults in our population which was a novelty in our report. We could not explain exactly the causality and pathomechanism of why BW and IR had the best predictor value. Further research is needed to confirm it. We found that the cut-off values of BMI and WC were 24.93 kg/m^2^ and 91.5 cm respectively, which nearly met the criteria of obesity classified by World Health Organization for the Asian population (BMI = 25 kg/m^2^) and criteria for abdominal obesity (WC = 90 cm). From this result, we suggest that young male adults who meet the cut-off point for the obesity index mentioned above should be screened for risk of IR and further the cardiometabolic disorders to prevent the development of diseases. In our study, the BF and VF had a slightly larger AUC than WC and BMI. The possible explanation might be that the BF and VF in male adults were better in reflecting adipose tissue, one of the main causes of IR, while WC and BMI did not exactly mirror the fat content.

The WC and BMI have been used as the traditional obesity index for assessing IR. Bluher [14] reported that BMI and WC were best predictors of cardiometabolic comorbidities in obese pubertal adolescents. Ling [15] reported that BMI and WC were simple predictors of fasting insulin and insulin resistance in overweight and obese adolescents. The WC was strongly associated with glucose and lipid disturbance in obese subjects [16]. The BMI was strongly correlated with HOMA-IR in adolescents [17]. In the past few years, several obesity indexes have assessed using the BIA method which included evaluating the BF and VF. Fernandes [18] found that the BIA method was good in identifying excess visceral and subcutaneous fat. Fat mass measured by the BIA method was associated with the fat mass evaluated from Dual Energy X-ray Absorptiometry (DXA) [19]. A study of Korean high school students showed that the BF was associated with HOMA-IR in male students [20].

Our result and other previous results showed that predictions of IR might be influenced by ethnicity, gender, and age. Our study is one of the few studies concerning young male adults in South East Asia, especially in Indonesia, which studies the association of five obesity indices with IR. Our study revealed that BW, BMI, WC, BF, and VF had a strong association with IR, but BW, BF, and VF had slightly better predictor value than WC and BMI in the healthy young male adult population. The clinical implication was that BW along with BF and VF measured by BIA could be used for predicting IR alongside the common obesity indices such as WC and BMI.

Our study had several limitations. Firstly, this was a cross-sectional study, therefore, causal relationships of obesity indices and IR could not be determined and explained. Secondly, the number of study subjects was limited, relatively small, and recruited from a single population. Thirdly, we used the BIA method for measuring the percentage of BF and VF level which was not the gold standard. This was, however, easy to perform, simple, cheap, and had good sensitivity (85%) and specificity (100%) compared to Dual Energy X-Ray Absorptiometry (DXA) [19]. The BIA devices are accurate in the estimation of body composition, especially fat-free mass (FFM), whereas the estimation of VF is under or overestimated by as much as −18% to +20.4% [21].

In conclusion, we demonstrated that BW, BMI, WC, BF, and VF had a significant correlation with IR but BW, BF and VF had a slightly better predictor value.

## Figures and Tables

**Table 1:** Characteristics of Total, IR and non-IR Groups.

Variable	Total (*n* = 140)	Non-IR (*n* = 105)	IR (*n* = 35)	*p*
Age, year	21 (18–25)	21 (18–25)	21 (18–24)	0.15 ^#^
BW, kg	66.45 (44.4–136.10)	64.2 (44.4–136.10)	85.7 (56–129.40)	0.00 ^#^
BMI, kg/m^2^	23.77 (13.14–49.9)	22.82 (16.31–49.99)	27.90 (13.14–49.31)	0.00 ^#^
WC, cm	85 (66–136)	83 (66–136)	99 (71–135)	0.00 ^#^
BF, %	21.18 ± 7.31	19.46 ± 6.66	26.33 ± 6.78	0.00 *
VF	7 (1–23)	6 (1–23)	11 (1–23)	0.00 ^#^
FPG, mg/dL	97.49 ± 8.56	96.86 ± 8.11	99.38 ± 9.64	0.131 *
Insulin, µIU/mL	11 (3.52–46.82)	9.21 (3.52–15.75)	21.16 (14.17–46.82)	0.00 ^#^
HOMA-IR	2.66 (0.68–14.22)	2.2 (0.68–3.74)	5.11 (3.75–14.22)	0.00 ^#^

Data are expressed by mean ± standard deviation for normally distributed variables and median (minimum–maximum) for non-normally distributed variables. Subjects are divided into two groups, IR negative and IR positive, based on HOMA-IR ≥75 percentile value (cut-off 3.75). IR = Insulin Resistance, BW = body weight, BMI = body mass index, WC = waist circumference, BF = body fat percentage, VF = visceral fat level, * T Test, ^#^ Mann Whitney Test.

**Table 2:** Correlation of IR with Several Indices of Obesity.

Variables	Correlation Coefficient *	*p*
BW	0.480	0.00
BMI	0.390	0.00
WC	0.456	0.00
BF	0.438	0.00
VF	0.438	0.00

IR = Insulin Resistance, BW = body weight, BMI = body mass index, WC = waist circumference, BF = body fat percentage, VF = visceral fat level * Spearman Correlation Test.

**Table 3:** The AUC, Sensitivity, and Specificity by the Most Optimal Cut-Off Point of Different Obesity Indices in Predicting IR.

Variables	AUC (95% CI)	Sensitivity	Specificity	Cut-Off Point
BW	0.788 (0.701–0.876)	0.714	0.733	70.2
BMI	0.747 (0.651–0.844)	0.714	0.733	24.93
WC	0.765 (0.666–0.864)	0.714	0.781	91.5
BF	0.779 (0.691–0.868)	0.743	0.714	22.05
VF	0.766 (0.675–0.858)	0.686	0.743	8.5

AUC = area under the ROC curve, IR = Insulin Resistance, BW = body weight, BMI = body mass index, WC = waist circumference, BF = body fat percentage, VF = visceral fat level.

**Table 4:** Logistic Regression Analysis for Determining IR.

Variables	*p*	OR	95% CI	
			Lower	Upper
BW	0.00	1.065	1.037	1.095
BMI	0.00	1.144	1.065	1.228
WC	0.00	1.076	1.041	1.112
BF	0.00	1.155	1.082	1.233
VF	0.00	1.227	1.116	1.349

IR = Insulin Resistance, BW = body weight, BMI = body mass index, WC = waist circumference, BF = body fat percentage, VF = visceral fat level.
